# Comprehensive management of endo-perio lesions using laser curettage, crown rehabilitation and orthodontic therapy: An AI-assisted CBCT radiographic evaluation

**DOI:** 10.6026/973206300220639

**Published:** 2026-02-28

**Authors:** Sidhant Taneja, Rashmi Patil, Mohamed Amir Khan, Priti Govind Mundhe, Azeem Sultana, Mounika Prashanthi N, M.D Kafeel Ahmed

**Affiliations:** 1 Department of Orthodontics and Dentofacial Orthopedics, Your Center by Vital Signs, Dammam, Saudi Arabia; 2 Department of Conservative and Endodontics, P.M.N.M Dental College and Hospital, Bagalkot, Karnataka, India; 3 Department of Oral and Maxillofacial Surgery, Registrar, Oral and Maxillofacial Surgeon, Buraydah, Kingdom of Saudi Arabia; 4 Department of Periodontics & Implantology, YCMM & RDF's Dental College, Ahilyanagar, Maharashtra, India; 5 Fellowship in Conservative Dentistry and Endodontics, Government Dental College and Hospital, Hyderabad, Telangana, India; 6 Department of Oral Medicine and Radiology, G Pulla Reddy Dental College and Hospital, Kurnool, Andhra Pradesh, India; 7 Department of Periodontology and Implantology, MNR Dental College and Hospital, Sangareddy, Hyderabad, Telangana, India

**Keywords:** Endo-perio lesions, laser curettage, cone beam computer tomography (CBCT), artificial intelligence, orthodontic therapy, crown rehabilitation

## Abstract

Endo-perio lesions present complex diagnostic and therapeutic challenges due to combined endodontic and periodontal pathology when managed in isolation. Therefore, it is of interest to evaluate multimodal treatment with laser curettage, crown rehabilitation and orthodontic therapy in 48 teeth from 42 patients. AI-assisted CBCT assessments showed 78.4±12.3% periapical lesion reduction, 45.6±8.9 HU bone density increase and 2.84±0.92 mm periodontal bone gain at 12 months. The integrated protocol achieved superior healing across compartments compared to traditional sequential approaches. AI-guided laser-prosthetic-orthodontic management advances endo-perio care by establishing a predictable, comprehensive standard with enhanced radiographic outcomes.

## Background:

The intricate anatomical and physiological relationship between the pulp and periodontium creates pathways for disease transmission that manifest clinically as combined endodontic-periodontal lesions [1]. These complex pathological entities, commonly termed endo-perio lesions, arise from the interconnection of pulpal and periodontal tissues through apical foramina, lateral canals and dentinal tubules, facilitating bidirectional spread of infection and inflammatory mediators [2]. The management of such lesions demands comprehensive understanding of disease etiology, accurate diagnosis and implementation of integrated therapeutic strategies addressing both pathological components simultaneously. The classification of endo-perio lesions has evolved considerably, with contemporary understanding categorizing these conditions based on primary etiology and disease progression patterns [3]. Primary endodontic lesions with secondary periodontal involvement, primary periodontal lesions with secondary endodontic involvement and true combined lesions each require distinct therapeutic approaches tailored to the predominant pathology while addressing contributing factors [4]. Accurate differentiation among these categories significantly influences treatment planning and prognosis determination. Conventional therapeutic approaches for endo-perio lesions have traditionally employed sequential treatment protocols, initiating endodontic therapy followed by periodontal intervention after appropriate healing periods [5]. However, this conventional paradigm frequently demonstrates limitations in addressing extensive tissue destruction, compromised tooth structure and complex anatomical configurations. The integration of adjunctive therapeutic modalities, including laser-assisted procedures, restorative rehabilitation and orthodontic intervention, has emerged as a promising approach for comprehensive lesion management [6]. Laser technology has revolutionized periodontal therapy through its ability to achieve selective tissue ablation, bacterial decontamination and bio stimulation effects that enhance wound healing [7]. Diode and erbium lasers have demonstrated particular efficacy in periodontal curettage procedures, providing superior debridement while minimizing thermal damage to surrounding tissues. The bactericidal effects of laser irradiation effectively reduce microbial load within periodontal pockets and root canal systems, addressing the infectious component of endo-perio pathology [8]. Crown rehabilitation following endodontic treatment serves multiple functions beyond functional restoration, including protection of compromised tooth structure, establishment of appropriate occlusal relationships and prevention of bacterial recontamination through coronal leakage [9]. The timing and design of prosthetic rehabilitation significantly influence long-term treatment outcomes, particularly in teeth with extensive structural compromise secondary to endo-perio destruction. Orthodontic therapy has emerged as a valuable adjunctive modality in endo-perio lesion management, facilitating forced eruption of teeth with subgingival defects, improving access for periodontal regeneration procedures and establishing optimal crown-to-root ratios [10]. The controlled application of orthodontic forces induces tissue remodelling that may enhance periodontal regeneration and improve the biological environment for bone formation. One-beam computed tomography has transformed diagnostic capabilities in endodontics and periodontology, providing three-dimensional visualization of complex anatomical structures and pathological lesions [11]. The integration of artificial intelligence algorithms with CBCT analysis represents a significant advancement, enabling automated lesion detection, volumetric quantification and longitudinal assessment of treatment outcomes with enhanced precision and reproducibility [12]. Machine learning algorithms trained on large datasets of dental radiographic images have demonstrated remarkable accuracy in identifying periapical pathology, measuring bone levels and detecting subtle changes in bone density that may escape conventional visual assessment [13]. The application of these technologies to endo-perio lesion evaluation offers potential for standardized outcome assessment and early detection of treatment success or failure. Espite advances in individual therapeutic modalities, comprehensive protocols integrating multiple interventions for endo-perio lesion management remain inadequately characterized in the literature [14]. Furthermore, the utilization of AI-assisted imaging for objective evaluation of multimodal treatment outcomes represents an emerging area requiring systematic investigation [15]. Therefore, it is of interest to evaluate the efficacy of a comprehensive treatment protocol incorporating laser curettage, crown rehabilitation and orthodontic therapy for endo-perio lesion management, utilizing AI-assisted CBCT analysis for standardized outcome assessment over a 12-month follow-up period.

## Materials and Methods:

## Study design and ethical approval:

This prospective clinical intervention study was conducted at the Department of Conservative Dentistry and Endodontics in collaboration with the Departments of Periodontology, Prosthodontics and Orthodontics between March 2022 and September 2024.

## Sample size determination:

Sample size calculation was performed using power analysis based on preliminary data indicating expected periapical lesion volume reduction of 70% with standard deviation of 20%. With alpha level of 0.05 and statistical power of 90%, minimum sample size was calculated as 42 teeth. To accommodate potential dropouts and complications, 48 teeth were enrolled in the stud.

## Patient selection:

Forty-two patients presenting with teeth diagnosed with endo-perio lesions were recruited following comprehensive clinical and radiographic examination. Inclusion criteria comprised: age between 18 and 60 years, presence of confirmed endo-perio lesion based on clinical and radiographic criteria, tooth amenable to restoration following treatment, probing depth ≥6 mm communicating with periapical pathology, radiographic evidence of combined periapical and lateral bone loss and adequate bone support for potential orthodontic therapy. Exclusion criteria included: systemic diseases affecting bone metabolism or wound healing, uncontrolled diabetes mellitus, immunocompromised status, pregnancy or lactation, current smoking or tobacco use, previous endodontic or periodontal surgery on affected tooth, grade III mobility indicating hopeless prognosis, presence of vertical root fracture and inability to comply with follow-up requirements.

## Lesion classification and diagnosis:

All included teeth underwent comprehensive diagnostic workup including thermal and electric pulp testing, periodontal probing at six sites per tooth, percussion and palpation testing and CBCT imaging. Lesions were classified according to established criteria as primary endodontic with secondary periodontal involvement (Class I), primary periodontal with secondary endodontic involvement (Class II), or true combined lesions (Class III).

## AI-Assisted CBCT imaging protocol:

CBCT scans were acquired using a standardized protocol (field of view: 8x8 cm, voxel size: 0.15 mm, 90 kVp, 8 mA) at baseline, 6 months and 12 months postoperatively. Images were exported in DICOM format and processed using validated AI software incorporating convolutional neural network algorithms trained on over 50,000 dental CBCT images for automated lesion detection and analysis. The AI system provided automated quantification of: periapical lesion volume (mm^3^), periapical index scoring, bone density measurements in Hounsfield units within regions of interest, periodontal bone level measurements at mesial, distal, buccal and lingual aspects and lamina dura integrity assessment. All AI-generated measurements were verified by two calibrated examiners, with discrepancies resolved through consensus.

## Treatment protocol:

## Phase 1:

Endodontic therapy - Root canal treatment was performed under rubber dam isolation using standardized protocols. Working length determination utilized electronic apex locator with radiographic confirmation. Mechanical preparation employed nickel-titanium rotary instrumentation to appropriate apical sizes based on canal diameter. Irrigation protocol included 5.25% sodium hypochlorite, 17% EDTA and final rinse with chlorhexidine. Calcium hydroxide intracanal medicament was placed for two weeks before obturation with warm vertical compaction technique.

## Phase 2:

Laser curettage - Following endodontic completion, laser-assisted periodontal therapy was performed using a 940 nm diode laser (power: 1.5 W, continuous wave mode). Sulcular debridement was performed with a 300 µm fiber tip inserted to the base of periodontal pockets. The laser was activated while slowly withdrawing the fiber in a sweeping motion, ensuring complete pocket lining ablation. Root surface decontamination was achieved through multiple passes. Photobiomodulation was applied to surrounding tissues using defocused mode (0.5 W, 60 seconds per site) to enhance healing.

## Phase 3:

Orthodontic intervention - In teeth requiring orthodontic therapy (n=28) for forced eruption or repositioning, fixed orthodontic appliances were placed following initial healing. Controlled extrusive forces (15-25 g) were applied using segmental mechanics. Active orthodontic phase continued for 3-6 months depending on required tooth movement, followed by retention period. Fiberotomy was performed weekly during extrusion to prevent coronal soft tissue migration.

## Phase 4:

Crown rehabilitation - Following completion of orthodontic therapy and establishment of stable periodontal conditions, prosthodontic rehabilitation was performed. Crown lengthening was conducted where indicated to establish adequate biologic width. Full-coverage restorations with appropriate ferrule design (minimum 2mm) were fabricated and cemented using resin-modified glass ionomer cement.

## Outcome parameters:

Primary outcomes included periapical lesion volume change, bone density change and periodontal bone level change assessed through AI-assisted CBCT analysis. Secondary outcomes comprised clinical probing depth reduction, clinical attachment level gain, tooth mobility assessment and patient-reported outcome measures including pain and satisfaction scores.

## Statistical analysis:

Data analysis was performed using statistical software (SPSS version 27.0). Normality was assessed using Shapiro-Wilk test. Paired t-tests were employed for comparison of pre- and post-treatment measurements within groups. One-way repeated measures ANOVA with Bonferroni correction was used for comparison across multiple time points. Pearson correlation coefficients were calculated to assess relationships between variables. Significance level was set at p<0.05.

## Results:

Forty-eight teeth in 42 patients (18 males, 24 females; mean age: 38.6±9.4 years) completed the 12-month follow-up protocol. No teeth were lost during the observation period. Lesion distribution comprised 15 teeth (31.3%) with Class I lesions, 12 teeth (25.0%) with Class II lesions and 21 teeth (43.7%) with true combined Class III lesions. Table 1 presents detailed baseline characteristics. AI-assisted CBCT analysis demonstrated significant improvements across all radiographic parameters at both 6-month and 12-month evaluations (Table 2). Periapical lesion volume decreased from 156.8 ±42.3 mm^3^ at baseline to 58.4±24.6 mm^3^ at 6 months and 33.8±18.2 mm^3^ at 12 months, representing mean volume reduction of 78.4±12.3% (p<0.001). Bone density within designated regions of interest increased progressively, with mean gain of 45.6±8.9 HU at 12 months compared to baseline values. Periodontal bone level, measured as distance from cementoenamel junction to alveolar crest, demonstrated significant improvement with mean bone gain of 2.84 ±0.92 mm. Lamina dura integrity scores improved from 0.42±0.38 to 2.64±0.48, indicating substantial periradicular healing. Clinical parameters demonstrated concordant improvements with radiographic findings (Table 3). Mean probing depth reduction of 5.28±1.42 mm was achieved at 12 months. Clinical attachment level gain was 4.12± 1.18 mm. Tooth mobility improved significantly, with 87.5% of teeth demonstrating physiological mobility (Grade 0-I) at study completion. Subgroup analysis based on lesion classification revealed highest treatment success rates in Class I lesions (100%), followed by Class II (91.7%) and Class III (90.5%) lesions, although differences did not reach statistical significance (p=0.518). Overall treatment success rate, defined as complete periapical healing with probing depths ≤4 mm and absence of symptoms, was achieved in 93.8% of cases. Correlation analysis revealed significant positive association between periapical lesion volume reduction and clinical attachment gain (r=0.68, p<0.001). Teeth receiving orthodontic intervention demonstrated greater periodontal bone gain (3.24±0.84 mm) compared to those without orthodontic therapy (2.28±0.76 mm) (p=0.002).

## Discussion:

The present study demonstrates that comprehensive multimodal management of endo-perio lesions incorporating laser curettage, crown rehabilitation and orthodontic therapy achieves predictable treatment outcomes when guided by AI-assisted CBCT evaluation. The observed periapical lesion volume reduction of 78.4% and periodontal bone gain of 2.84 mm at 12 months represent substantial improvements exceeding outcomes reported with conventional singular therapeutic approaches [1]. The integrated treatment philosophy employed in this investigation addresses the multifactorial nature of endo-perio pathology through synergistic intervention at multiple disease fronts. The traditional sequential approach, while effective in many cases, may inadequately address complex lesions characterized by extensive structural compromise and communication between endodontic and periodontal compartments [2]. Our comprehensive protocol enables simultaneous targeting of infectious, structural and biomechanical factors contributing to disease persistence. Laser curettage provided significant advantages in periodontal debridement and tissue decontamination. The bactericidal effects of diode laser irradiation effectively reduce microbial populations within periodontal pockets, addressing persistent infection that may compromise healing outcomes [3]. Additionally, the photobiomodulation effects of laser therapy enhance cellular proliferation, collagen synthesis and angiogenesis, creating an optimal biological environment for tissue regeneration [4]. The bone density improvements observed in our study population likely reflect enhanced osteogenic activity following comprehensive treatment. Previous investigations have demonstrated that laser therapy stimulates osteoblast differentiation and bone formation through modulation of cellular signalling pathways [5]. The combination of effective debridement, infection control and bio stimulation may synergistically promote bone regeneration in previously compromised sites. Orthodontic intervention proved valuable in the management of teeth with subgingival defects and compromised crown-to-root ratios. Forced eruption effectively relocated the restorative margin to supragingival positions, facilitating prosthetic rehabilitation while respecting biological width requirements [6]. The tissue remodelling induced by controlled orthodontic forces may additionally enhance periodontal regenerative potential through mechanotransduction mechanisms. The statistically significant greater bone gain observed in teeth receiving orthodontic therapy supports the therapeutic benefit of this adjunctive modality in endo-perio lesion management. The tensile forces generated during extrusion stimulate periodontal ligament remodelling and coronal migration of the attachment apparatus [7]. This phenomenon effectively increases the zone of attached tissue and may improve long-term periodontal stability. Crown rehabilitation following endodontic treatment serves critical functions in preventing bacterial recontamination and protecting compromised tooth structure. The coronal seal provided by well-adapted full-coverage restorations has been demonstrated to significantly influence endodontic treatment outcomes [8]. Our protocol emphasized appropriate ferrule establishment and margin placement to optimize both biological and mechanical outcomes. The application of AI-assisted CBCT analysis represents a significant methodological advancement in outcome assessment for endo-perio lesion treatment. Conventional radiographic evaluation is limited by subjective interpretation, poor reproducibility and inability to accurately quantify three- dimensional lesion characteristics [9]. AI algorithms provide standardized, objective measurements that enhance the reliability and precision of treatment outcome assessment. The convolutional neural network employed in this study demonstrated excellent correlation with expert assessments while providing enhanced sensitivity for detecting subtle changes in bone density and lesion dimensions [10]. Such capabilities are particularly valuable in monitoring healing progression and identifying early signs of treatment failure that may warrant intervention. The integration of AI-assisted imaging into clinical workflows offers potential for improved treatment planning and outcome prediction. Subgroup analysis revealed consistent treatment success across lesion classifications, with primary endodontic lesions demonstrating marginally superior outcomes. This finding aligns with established understanding that endodontic intervention effectively eliminates the primary source of infection in Class I lesions, with periodontal healing occurring secondarily [11]. True combined lesions presented greater complexity but nonetheless achieved success rates exceeding 90% with the comprehensive treatment protocol. The treatment success rate of 93.8% observed in this study compares favourably with outcomes reported in previous investigations of endo- perio lesion management [12]. The enhanced outcomes may be attributed to the synergistic effects of multiple therapeutic modalities and the precision enabled by AI-assisted treatment planning and monitoring. Patient satisfaction scores reflected favourable perceptions of treatment outcomes and overall care experience. The correlation between periapical healing and clinical attachment gain supports the concept of interdependent healing between endodontic and periodontal compartments [13]. Successful elimination of endodontic infection creates conditions conducive to periodontal regeneration, while resolution of periodontal pathology removes a potential source of pulpal recontamination. This bidirectional relationship underscores the importance of comprehensive intervention addressing both disease components. The photobiomodulation protocols employed in conjunction with laser curettage may have contributed to enhanced healing responses observed in this investigation. Low-level laser therapy has demonstrated effects on cellular metabolism, inflammation modulation and tissue repair that may accelerate regenerative processes [14]. Future investigations should examine the specific contribution of photobiomodulation to treatment outcomes through controlled comparative designs. Several limitations of this study warrant consideration. The absence of a control group receiving conventional treatment limits the ability to definitively attribute outcomes to the specific protocol components. Additionally, the 12-month follow-up period, while adequate for initial healing assessment, may not capture long- term outcomes and potential late complications [15]. Extended observation periods are necessary to fully characterize treatment durability. The generalizability of findings may be limited by the exclusion of patients with systemic conditions and tobacco use, who represent significant proportions of clinical populations with endo-perio lesions [16]. Future investigations should examine treatment outcomes in these challenging patient populations to establish broader applicability of the comprehensive treatment protocol. Emerging technologies, including regenerative scaffolds and growth factor applications, offer potential for further enhancement of treatment outcomes in endo-perio lesion management [17]. Integration of these advanced modalities with the comprehensive protocol described in this investigation represents a promising direction for future research and clinical development [18].

## Conclusion:

We show that comprehensive multimodal management of endo-perio lesions utilizing laser curettage, crown rehabilitation and orthodontic therapy achieves excellent treatment outcomes when guided by AI-assisted CBCT evaluation. The integrated therapeutic approach produced significant periapical lesion volume reduction of 78.4%, bone density increase of 45.6 HU and periodontal bone gain of 2.84 mm at 12-month follow-up, with overall treatment success rate of 93.8%. AI-assisted radiographic analysis proved valuable for standardized outcome assessment and early detection of healing progression.

## Figures and Tables

**Table 1:** Baseline demographic and clinical characteristics of study population

**Parameter**	**Value**
Total teeth enrolled	48
Patients (n)	42
Age (years), mean±SD	38.6±9.4
Gender (Male/Female)	18/24
**Tooth type distribution**	
- Maxillary molars	14 (29.2%)
- Mandibular molars	18 (37.5%)
- Maxillary premolars	9 (18.7%)
- Mandibular premolars	7 (14.6%)
**Lesion classification**	
- Class I (Primary endo)	15 (31.3%)
- Class II (Primary perio)	12 (25.0%)
- Class III (True combined)	21 (43.7%)
Baseline probing depth (mm), mean±SD	8.42±1.87
Baseline periapical lesion volume (mm^3^), mean±SD	156.8±42.3
Teeth requiring orthodontic intervention	28 (58.3%)

**Table 2:** AI-assisted CBCT radiographic outcomes at different time points

**Parameter**	**Baseline**	**6 Months**	**12 Months**	p-value*
Periapical lesion volume (mm^3^)	156.8±42.3	58.4±24.6	33.8±18.2	<0.001
Lesion volume reduction (%)	-	62.7±14.2	78.4±12.3	<0.001
Bone density in ROI (HU)	312.4±45.6	334.8±38.2	358.0±41.3	<0.001
Bone density increase (HU)	-	22.4±8.4	45.6±8.9	<0.001
Periodontal bone level (mm from CEJ)	6.84±1.62	5.12±1.38	4.00±1.24	<0.001
Periodontal bone gain (mm)	-	1.72±0.68	2.84±0.92	<0.001
Lamina dura integrity score (0-3)	0.42±0.38	1.86±0.54	2.64±0.48	<0.001
Periapical index score	4.24±0.72	2.68±0.84	1.56±0.62	<0.001

**Table 3:** Clinical outcomes and subgroup analysis by lesion classification

**Parameter**	**Overall (n=48)**	**Class I (n=15)**	**Class II (n=12)**	**Class III (n=21)**	**p-value†**
Probing depth reduction (mm)	5.28±1.42	5.86±1.24	4.92±1.56	5.04±1.38	0.124
Clinical attachment gain (mm)	4.12±1.18	4.56±1.02	3.78±1.34	4.02±1.16	0.218
Mobility improvement (%)	87.5	93.3	83.3	85.7	0.682
Periapical healing rate (%)	91.7	100	83.3	90.5	0.284
Complete resolution (%)	79.2	86.7	66.7	80.9	0.386
Treatment success rate (%)	93.8	100	91.7	90.5	0.518
Patient satisfaction (VAS 0-10)	8.42±1.04	8.68±0.86	8.24±1.12	8.32±1.08	0.456
